# In-depth analysis of research hotspots and emerging trends in AI for retinal diseases over the past decade

**DOI:** 10.3389/fmed.2024.1489139

**Published:** 2024-11-20

**Authors:** Mingkai Guo, Di Gong, Weihua Yang

**Affiliations:** ^1^The Third School of Clinical Medicine, Guangzhou Medical University, Guangzhou, China; ^2^Shenzhen Eye Institute, Shenzhen Eye Hospital, Jinan University, Shenzhen, China

**Keywords:** artificial intelligence, retinal disease, deep learning, machine learning, hotspot, trend

## Abstract

**Background:**

The application of Artificial Intelligence (AI) in diagnosing retinal diseases represents a significant advancement in ophthalmological research, with the potential to reshape future practices in the field. This study explores the extensive applications and emerging research frontiers of AI in retinal diseases.

**Objective:**

This study aims to uncover the developments and predict future directions of AI research in retinal disease over the past decade.

**Methods:**

This study analyzes AI utilization in retinal disease research through articles, using citation data sourced from the Web of Science (WOS) Core Collection database, covering the period from January 1, 2014, to December 31, 2023. A combination of WOS analyzer, CiteSpace 6.2 R4, and VOSviewer 1.6.19 was used for a bibliometric analysis focusing on citation frequency, collaborations, and keyword trends from an expert perspective.

**Results:**

A total of 2,861 articles across 93 countries or regions were cataloged, with notable growth in article numbers since 2017. China leads with 926 articles, constituting 32% of the total. The United States has the highest h-index at 66, while England has the most significant network centrality at 0.24. Notably, the University of London is the leading institution with 99 articles and shares the highest h-index (25) with University College London. The National University of Singapore stands out for its central role with a score of 0.16. Research primarily spans ophthalmology and computer science, with “network,” “transfer learning,” and “convolutional neural networks” being prominent burst keywords from 2021 to 2023.

**Conclusion:**

China leads globally in article counts, while the United States has a significant research impact. The University of London and University College London have made significant contributions to the literature. Diabetic retinopathy is the retinal disease with the highest volume of research. AI applications have focused on developing algorithms for diagnosing retinal diseases and investigating abnormal physiological features of the eye. Future research should pivot toward more advanced diagnostic systems for ophthalmic diseases.

## 1 Introduction

Artificial Intelligence (AI), first introduced in 1956 (1), signifies machines undertaking tasks traditionally aligned with human intelligence. With its ability to process complex data, AI has become a transformative technology in healthcare, offering new solutions for early disease detection and personalized treatment. With AI encompassing Machine Learning (ML) and Deep Learning (DL), the latter advances by employing multilayer artificial neural networks to decode complex patterns from extensive data sets, facilitating the management of intricate diseases (2, 3). By 2015, DL models (4–6) had achieved human-level accuracy in image recognition tasks, underscoring AI’s potential to rival human capability in medicine and science. This prowess, particularly in tasks like image classification through ML algorithms (7–9), has garnered considerable focus. The evolution of digital imaging, image processing, and computer vision notably enhances the role of ML in autonomously identifying retinal maladies from color fundus photographs. Transfer learning (10, 11), as evidenced by prior studies, emerges as a potent strategy, especially under data scarcity, to mitigate overfitting and heighten model precision. Deep transfer learning, sidestepping the need for extensive manual annotations or a vast corpus of labeled training data, presents a more economical and efficient alternative to traditional image recognition methods (12).

Retinal diseases, significant contributors to severe vision impairment, encompass a spectrum from retinopathy to chorioretinopathy (13), with risk factors including age (14), myopia (15), diabetes (16), trauma (17), retinal vascular occlusion (18), hypertension (19), retinitis (20), and genetic disposition (21). The cruciality of early screening and diagnosis stands in preventing irreversible vision loss against the backdrop of widespread blindness and low vision issues amplified by the deficit of medical examination tools (22–24), specialist ophthalmologists (25–27), and effective interventions (28, 29), particularly in under-resourced areas (30). As AI-driven tools gain traction in retinal diagnostics, they support healthcare providers in regions with limited resources by enabling timely detection and intervention.

Recent endeavors have seen researchers leveraging ML and DL for AI-assisted diagnostic systems based on retinal imagery like color fundus photography or Optical Coherence Tomography (OCT), aiming at retinal disease screening. These innovations, rivaling ophthalmologist diagnostics, primarily target conditions such as Diabetic Macular Edema (DME) (31–33), Age-Related Macular Degeneration (AMD) (14, 34, 35), and even non-retinal diseases like glaucoma (36–38) through retinal imaging, despite fewer studies delving into comprehensive disease spectra (39, 40) or delineating normal from abnormal fundus images in AI-supported diagnosis (12, 41).

Our research, using bibliometric tools like CiteSpace, VOSviewer, and bibliometric.com, explores AI’s global impact on retinal disease management. This study aims to elucidate trends, geographical distribution, institutional contributions, and the emerging research focus areas within AI-driven retinal diagnostics. By applying bibliometric and manual screening, this research offers insights into the most influential studies and regions, advancing the understanding of AI’s current and future role in retinal disease management. The results from this analysis aim to guide AI and ophthalmology professionals, marking a significant leap toward integrating AI in retinal disease detection and management.

## 2 Methods

### 2.1 Selection of published data

On January 23, 2024, we retrieved citation data published between January 1, 2014, and December 31, 2023, from the Web of Science Core Collection (WoSCC) database. The data retrieval and verification were independently conducted by two authors to ensure accuracy and consistency.

The search formula used in the literature search was:

Topic = (“Artificial Intelligence” or “AI” or “transfer learning” or “neural network” or “Deep Learning” or “Robotic*” or “Supervised Learning” or “Unsupervised Learning” or “Computer Vision System” or “Computational Intelligence” or “Machine Learning” or “Evolutionary Computation” or “Ensemble Learning” or “Reinforcement Learning”) AND Topic = (“Retin*” or “Epiretinal” or “macular” or “Epiretinal Membrane”).

Only articles and review articles in English were included, while early access papers, conference proceedings, book chapters, data papers, and retracted articles were excluded. To refine the results, we manually screened each article title and abstract for relevance, with specific criteria for exclusion:

1. Studies that did not focus on eye diseases.
2. Studies that did not employ AI-based methods.

This approach allowed us to retain only those records directly relevant to AI applications in retinal diseases, enhancing the study’s specificity and accuracy.

**Figure 1:**
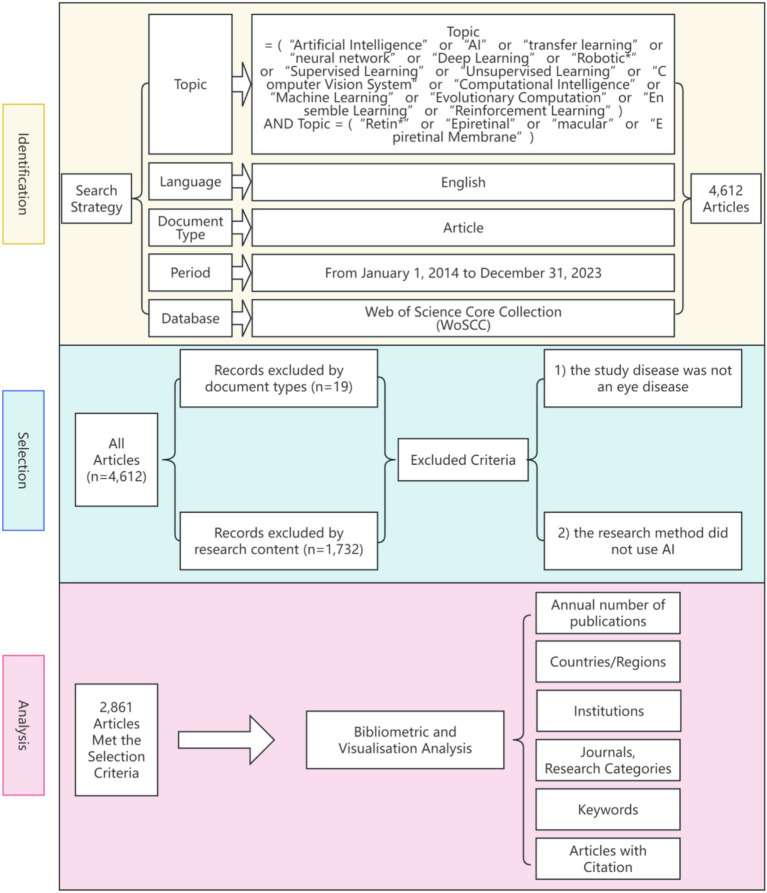
Frame flow diagram showing the detailed selection criteria and bibliometric analysis steps.

For each selected article, we extracted and analyzed essential bibliographic information, including title, publication year, country/region, institution, journal, and keywords. Based on this data, we conducted a thorough bibliometric analysis to explore global research hotspots in AI applications for retinal disease. A detailed search and data processing workflow is illustrated in Figure 1.

### 2.2 Research and analysis methods

CiteSpace 6.2 was used to perform a cluster analysis of countries or regions, organizations, journals, research categories, and keywords. Centrality was produced using CiteSpace. It represents the degree of cooperation between different regions or institutions. WoSCC can calculate the h-index of published literature. The h-index is defined as the maximum value of h such that the given author/journal has published at least h articles that have each been cited at least h times, which is a new method for evaluating academic achievements (42).

In addition to the functions provided by CiteSpace, we employed WOC Citation Topic Micro to identify and quantify AI applications across various retinal diseases. By counting keyword frequencies in the collected literature, CiteSpace and WOC Citation Topic Micro together facilitated the identification of major research focuses within the field of AI-assisted retinal diagnostics.

Using the bibliometric platform https://bibliometric.com/app, we classified the countries and institutions represented in the literature, generating comprehensive statistics on research distribution. VOSviewer generates heat maps based on keywords, which represent research hotspots. We further conducted an in-depth interpretation and comprehensive analysis of the included articles, especially the high-impact articles.

## 3 Results

### 3.1 Distribution of articles by publication year

**Figure 2:**
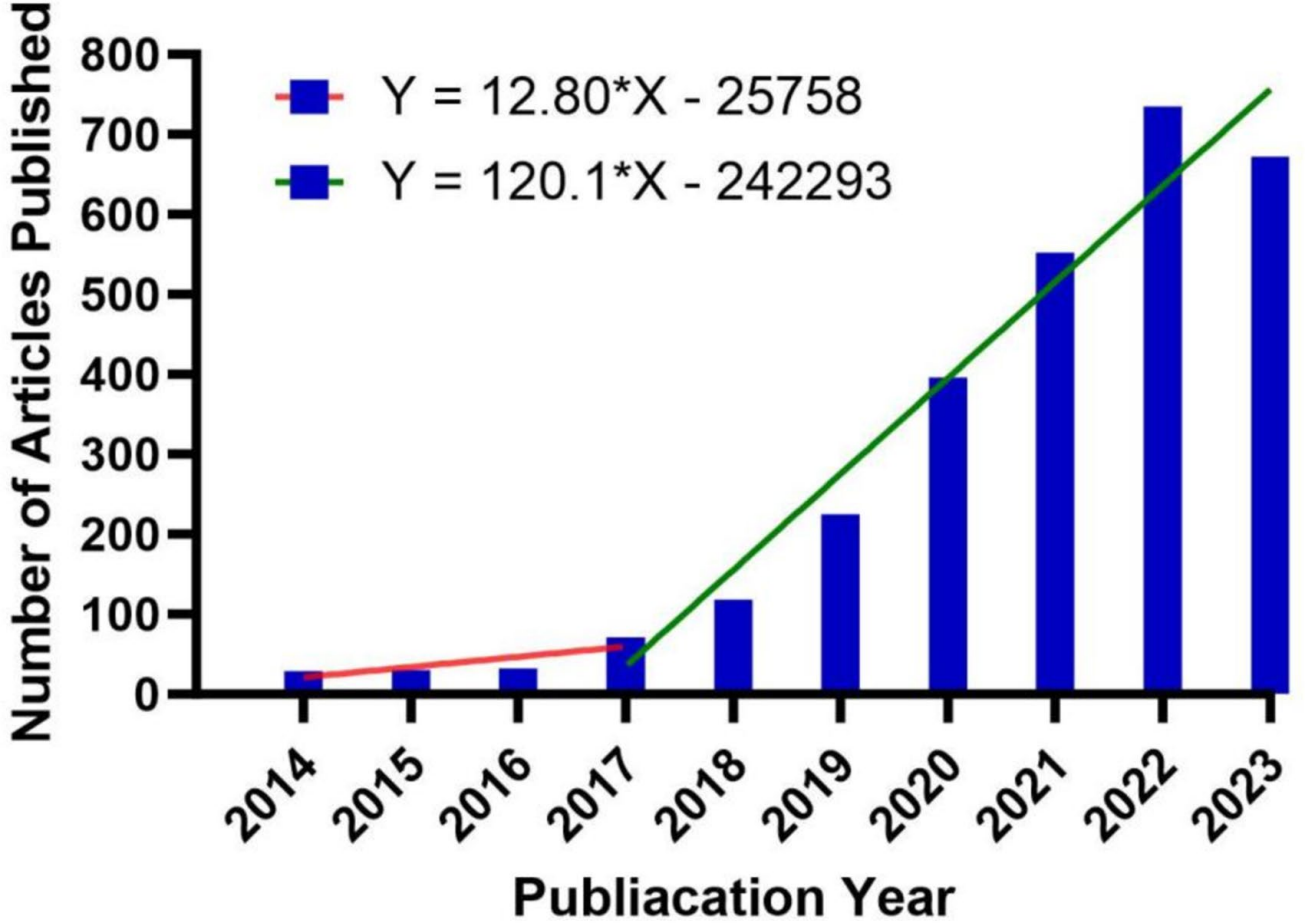
Annual number of articles on AI in retinal disease from 2014 to 2023.

We carried out an analysis of a total of 2,861 articles published from 2014 to 2023 on the subject of AI in retinal diseases. Employing the citation analyzing features of the WoSCC database, we computed the annual number of citations, with CiteSpace’s deduplication function ensuring the accuracy of these figures. As depicted in Figure 2, the publication frequency was modest before 2017, not exceeding 100 articles per year, and the annual article count’s trend line had a slope of 12.8, indicative of an average annual growth of about 12 articles. Post-2017, the yearly output surpassed 100 articles, showing a marked acceleration in recent years. The trend line’s slope steepened to 120.1, signaling an annual increase that averaged around 120 articles.

### 3.2 Countries or regions

**Figure 3:**
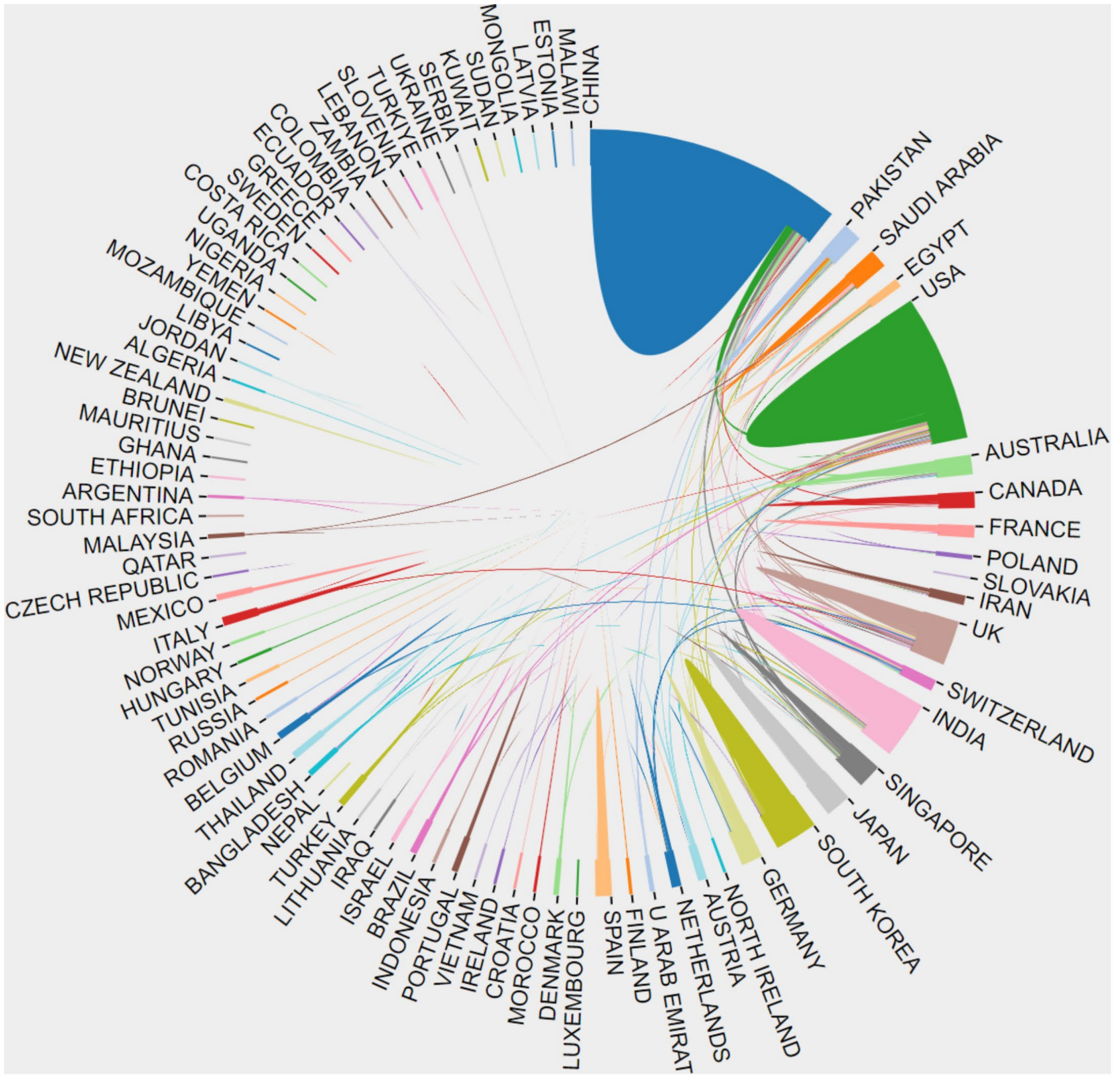
The cooperation of countries or regions that contributed to articles on AI in retinal diseases from 2014 to 2023.

The citation analyzer of the WoSCC database calculates the article output of various countries or regions, while CiteSpace facilitates the analysis of interdependencies among these entities. A total of 93 countries or regions are represented in the data. Figure 3 visually depicts the article output of each country and region, along with their collaborative engagements. The size of the labels within the circle reflects the number of articles from each entity, with China (926), the United States (637), and India (464) having the most significant shares. The interconnected lines between country labels in Figure 3 symbolize the collaborative efforts, where the density of these lines indicates the extent of literature exchange among countries.

**Figure 4:**
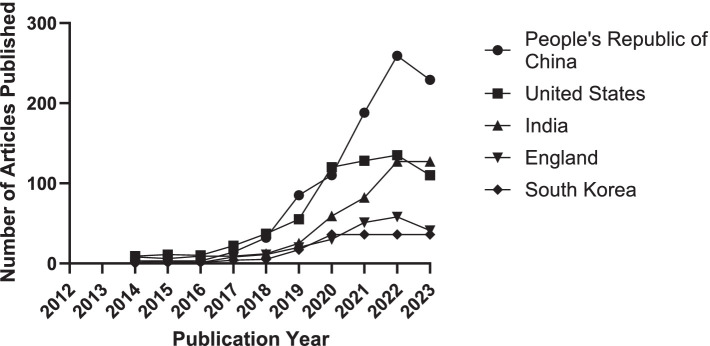
Trends in the number of articles contributed by the top 5 countries or regions from 2014 to 2023.

Further analysis reveals trends over the past decade regarding the article counts from the top five contributing countries or regions, as illustrated in Figure 4. Concurrently, Table 1 details the annual article counts on AI applications in retinal disease for these leading contributors.

**Table 1:** Top 5 countries or regions with articles on AI in retinal disease from 2014 to 2023.

Year	Count				
	People’s Republic of China	United States	India	England	South Korea
2014	3	9	8	1	0
2015	3	11	6	3	1
2016	3	10	9	1	1
2017	14	22	9	8	4
2018	32	37	12	11	5
2019	85	55	25	20	17
2020	110	120	59	30	36
2021	188	128	82	51	36
2022	259	135	127	58	36
2023	229	110	127	41	36

**Figure 5:**
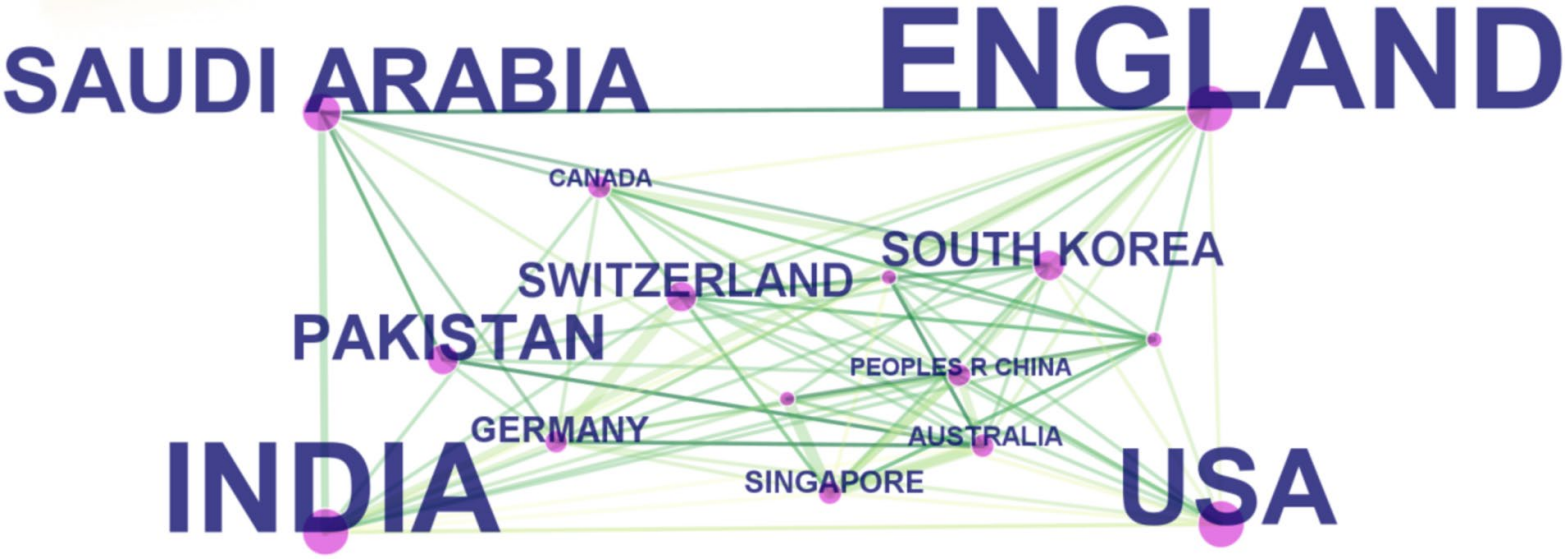
The cooperation of countries or regions that contributed to articles on applying artificial intelligence to the study of retinal diseases from 2014 to 2023.

The visual representation in Figure 5, through varying label sizes and green node areas, highlights the number of articles from each country or region, with connection lines delineating their cooperative relationships. The centrality measure in Table 2, represented by the purple circle’s size, assesses the influence of articles from each country, with England’s purple circle (0.24) being notably the largest, indicating its pivotal role in international collaborations.

**Table 2:** Top 10 countries or regions with articles on AI in retinal disease from 2014 to 2023.

Rank	Count	Proportion (%)	Centrality	H-index
1. People’s Republic of China	926	32.37	0.04	62
2. United States	637	22.27	0.18	66
3. India	464	16.22	0.22	44
4. England	224	7.83	0.24	39
5. South Korea	172	6.01	0.08	34
6. Saudi Arabia	135	4.72	0.13	22
7. Germany	133	4.65	0.05	31
8. Australia	126	4.40	0.06	33
9. Pakistan	106	3.70	0.10	22
10. Singapore	99	3.46	0.08	31

These findings are grounded in data displayed in Table 2, which also showcases the h-index, an indicator blending academic quality and impact. The United States boasts the highest h-index (66), denoting its leading influence. Summarily, from 2014 to 2023, China emerged as the most prolific publisher, England as the most collaborative, and the United States as having the most substantial influence in AI applications within retinal disease research.

### 3.3 Institutions

**Figure 6:**
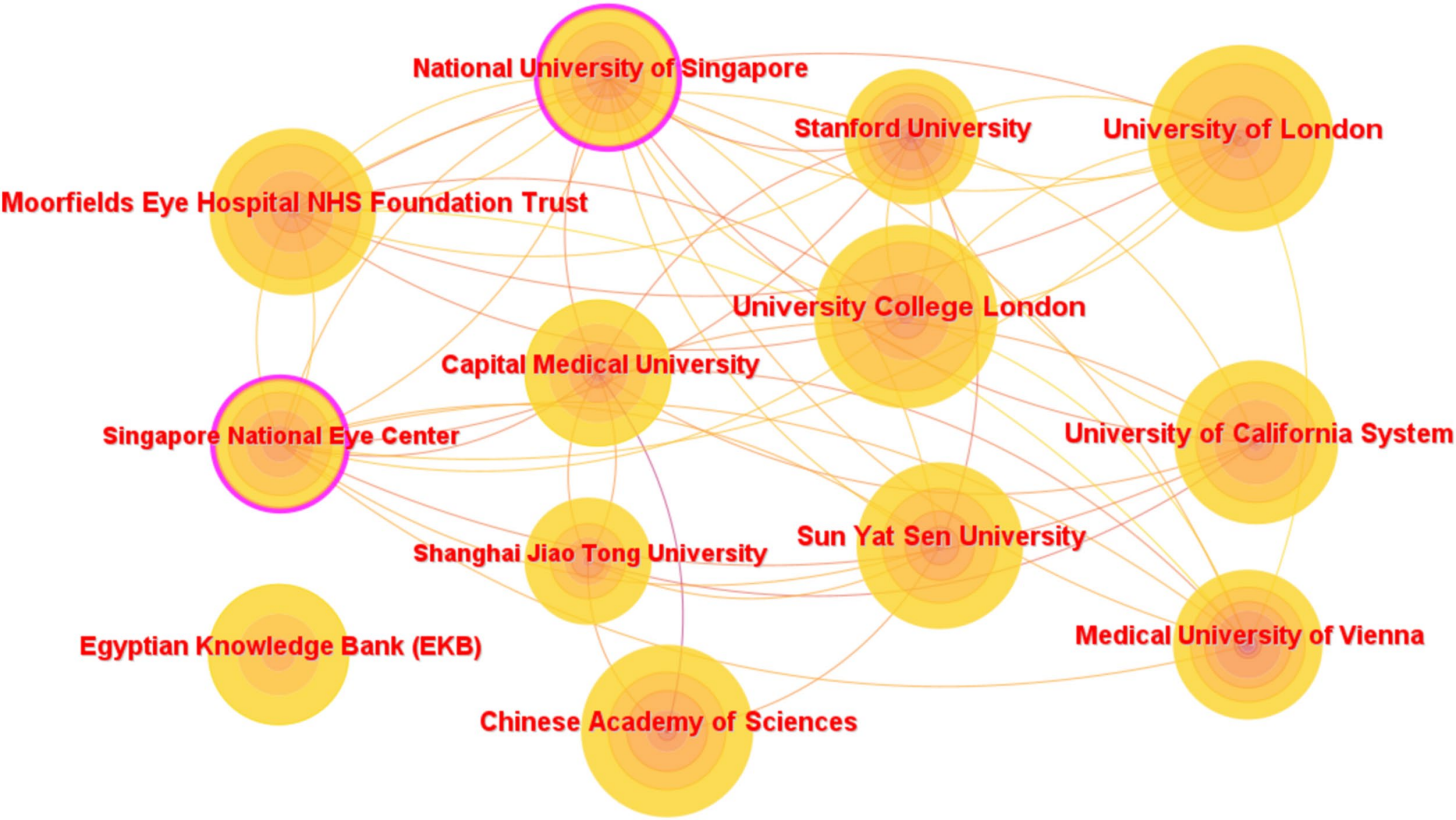
Network map of institutions that contributed to articles on AI in retinal diseases from 2014 to 2023.

The top 10 institutions with the most articles published are shown in Table 3. The data shown are the results of the CiteSpace software default settings. A total of 291 institutions were counted, constituting 1,375 partnerships. These include three institutions in the United Kingdom, three in China, and one in the United States, Austria, Egypt, and Singapore. Three of the top 5 h-index institutions are from the United Kingdom, and the other two are from China. The links between the labels in Figure 6 show the collaboration between agencies. The node size represents the number of articles sent. According to the centrality, the influence of the National University of Singapore was relatively high (0.13).

**Table 3:** Top 10 institutions with articles on AI in retinal disease from 2014 to 2023.

Rank	Countries/regions	Count	Centrality	h-index
1. University of London	England	99	0.05	25
2. University College London	England	95	0.05	25
3. Chinese Academy of Sciences	China	88	0.08	24
4. Moorfields Eye Hospital NHS Foundation Trust	England	81	0.06	23
5. Sun Yat-Sen University	China	79	0.05	21
6. University of California System	USA	78	0.09	20
7. Capital Medical University	China	65	0.03	19
8. Medical University of Vienna	Austria	65	0.07	24
9. Egyptian Knowledge Bank (EKB)	Egypt	62	0.05	16
10. National University of Singapore	Singapore	59	0.13	22

### 3.4 Journals and research categories

The knowledge foundation for the cited articles is the literature found in the cited articles. The research field of highly cited journals is a hotspot of attention. In the past 10 years, the knowledge-based research fields of artificial intelligence applied in ophthalmic diseases have included systems/computing/molecular/biology/genetics/health/nursing/medicine/ophthalmology, which constitute mathematics/systems/mathematics/neurology/hot topics at the forefront of sports/ophthalmology research. Table 4 lists the subject categories of the top 10 cited journals/proceedings. The most common research fields for citing journals include engineering technology/computing. The discipline most involved in the extracted version was classified as medicine/ophthalmology.

**Table 4:** Top 10 categories of journals on AI in retinal diseases from 2014 to 2023.

Rank	Web of Science categories	Numbers	% of 2,861
1	Ophthalmology	552	19.29
2	Engineering Electrical Electronic	478	16.71
3	Engineering Biomedical	443	15.48
4	Computer Science Interdisciplinary Applications	325	11.36
5	Computer Science Information Systems	323	11.29
6	Computer Science Artificial Intelligence	302	10.56
7	Radiology Nuclear Medicine Medical Imaging	266	9.30
8	Multidisciplinary Sciences	230	8.03
9	Medicine General Internal	216	7.55
10	Mathematical Computational Biology	193	6.75

### 3.5 Keywords

**Figure 7:**
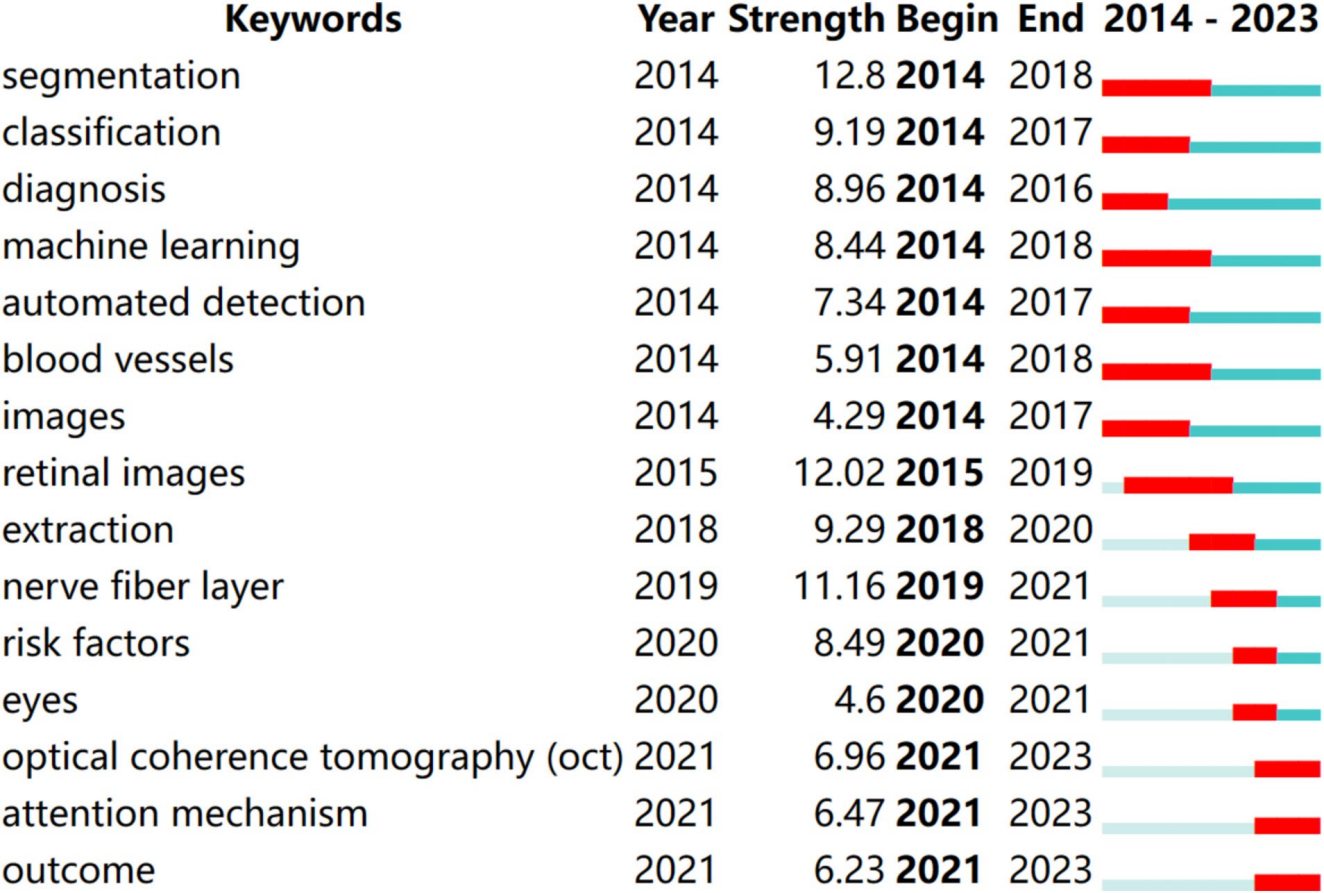
Top 10 keywords with the strongest citation bursts of articles on the application of artificial intelligence in retinal diseases from 2014 to 2023.

According to the keyword co-occurrence and cooperation network analysis chart, we analyzed new keywords used over the past 10 years to understand co-occurrence and cooperation. We modified the default settings of CiteSpace to “year per slice” = 1, “Top N%” = 10.0%, “Top N” = 30, and “minimum duration” = 2. The results are shown in Figure 7. Red indicates the emergence of keywords. The time trend was examined using the hotspot transfer method, which was applied to the first 15 keywords with the highest citation outbreak. From 2014 to 2023, the keywords that were consistently used included segmentation (2014–2018), classification (2014–2017), diagnosis (2014–2016), machine learning (2014–2018), automated detection (2014–2017), blood vessels (2014–2018), images (2014–2017), retinal images (2015–2019), extraction (2018–2020), nerve fiber layer (2019–2021), risk factors (2020–2021), eyes (2020–2021), OCT (2021–2023), attention mechanism (2021–2023), outcome (2021–2023).

These keywords highlight critical areas of research within AI applications in ophthalmology, reflecting advancements in diagnostic capabilities and methodologies. For instance, terms like “segmentation” and “classification” are foundational to developing accurate AI models for analyzing retinal images, while “attention mechanism” signifies a shift toward more sophisticated approaches that enhance model performance in identifying relevant features in complex datasets.

### 3.6 High-impact articles

The research includes the 10 most cited articles on AI in retinal diseases from 2014 to 2023, as listed in Table 5. These articles indicate that AI research in retinopathy holds promise, yet they also highlight certain limitations in clinical implementation.

**Table 5:** The top 10 articles on AI in retinal diseases from 2014 to 2023.

Rank	Title	Times cited	Interpretation of the findings	Advantages	Limitations
1	Development and Validation of a Deep Learning Algorithm for Detection of Diabetic Retinopathy in Retinal Fundus Photographs (43)	3,585	The article presents findings from the application of deep learning to create an algorithm for the automated detection of diabetic retinopathy and diabetic macular edema in retinal fundus photographs	DL algorithms offer adjustable sensitivity and specificity, enabling customization for various clinical environments DL networks can learn from extensive datasets without requiring predefined lesion characteristics DL algorithms facilitate rapid result reporting, benefiting timely clinical diagnosis and decision-making	System accuracy may decline due to reliance on ophthalmologist consensus, affecting the detection of subtle findings The neural network autonomously learns features, potentially using unrecognized human features, but specific features remain unidentified Focused training on diabetic retinopathy and macular edema may cause oversight of other retinopathies
2	Identifying Medical Diagnoses and Treatable Diseases by Image-Based Deep Learning (102)	1,580	This study established a diagnostic tool based on a transfer learning framework that trains a neural network with a fraction of the data of conventional approaches, for the screening of patients with common treatable blinding retinal diseases	The model demonstrated high accuracy with limited training data Its diagnostic performance on retinal OCT images matched that of experienced clinicians The model’s success with chest X-rays suggests broad applicability in medical imaging The occlusion test provided transparency and insights into the decision-making process of the model	Transfer learning facilitates precise training on small datasets, but may underperform compared to models trained from scratch on vast OCT image datasets Lesion recognition by the system in occlusion tests is constrained by lesion size Model performance significantly relies on the weights of the pre-trained model Additional clinical validation is required to evaluate its practical utility and effect on patient outcomes
3	Clinically applicable deep learning for diagnosis and referral in retinal disease (103)	1,215	This study proposed a novel deep-learning architecture for a clinically heterogeneous set of three-dimensional OCT scans	Two test datasets with diverse retinal pathologies were used, reflecting real-world clinical diversity 3D modeling and segmentation in OCT scans allowed for extensive tissue type analysis The AI framework mimicked clinical decision-making by separating scan evaluations from referral decisions Segmentation outputs served both as clinical training aids and as tools for medical image interpretation learning These outputs enabled the quantification of retinal features and pathology measurements, valuable for visual outcome studies and clinical trials The framework accurately triaged scans, matching or surpassing the performance of retina specialists and optometrists	The framework’s segmentation map, labeling each pixel singularly, might lack comprehensive diagnostic information for some clinical pathways The diversity of OCT-diagnosable diseases and global referral practices exceed the study’s scope Randomized trials are needed to validate its clinical efficacy and patient outcome impact
4	Development and Validation of a Deep Learning System for Diabetic Retinopathy and Related Eye Diseases Using Retinal Images From Multiethnic Populations With Diabetes (104)	1,141	The DL approach shows good sensitivity and specificity for detecting DR and related eye diseases while examining retinal images from multiethnic cohorts of diabetes patients	The DL System’s performance was clinically comparable to existing models, as evaluated by professional graders Training and validation datasets were notably extensive, encompassing diverse racial and ethnic patient images Validation occurred within an active diabetic retinopathy screening program, mirroring real-world scenarios including lower-quality and ungradable images Its cost-effectiveness allows for a semi-automated screening process, combining AI with human review for flagged cases	The training set was not entirely developed based on retinal specialists’ grading for all images The DL System used multiple levels of representation to analyze retinal images without explicitly showing the actual diabetic retinopathy lesions Identifying diabetic macular edema solely from fundus photos might miss cases without clinical exams and OCT
5	CE-Net: Context Encoder Network for 2D Medical Image Segmentation (105)	1,028	The study employed a context encoder network (CE-Net) for 2D medical image segmentation, enhancing high-level and spatial information capture. It demonstrated superior performance over the original U-Net and other advanced methods	CE-Net surpassed U-Net and other leading methods in diverse medical image segmentation tasks With dense atrous convolution and residual multi-kernel pooling, it captured enhanced high-level features, improving accuracy CE-Net’s versatility suited multiple 2D segmentation tasks	The proposed CE-Net method was validated only on 2D images in the current study It required fine-tuning with manually annotated data, which is labor-intensive and potentially scarce
6	Prediction of cardiovascular risk factors from retinal fundus photographs via deep learning (106)	798	The study shows DL’s capability to predict cardiovascular risk factors, such as age, gender, smoking status, systolic blood pressure, and major adverse cardiac events, from retinal fundus photos with high accuracy, leveraging anatomical features such as the optic disk and blood vessels	DL applied to retinal fundus images can predict cardiovascular risk factors Its accuracy in forecasting major adverse cardiovascular events matches that of the established composite risk calculators calculator Consistency across two validation datasets indicates generalizability The findings could elucidate the impact of cardiovascular diseases or risks on retinal vasculature/optic disks	The system only utilized retinal fundus photographs with a 45° field of view The overall size of the dataset was relatively small for DL Key inputs for cardiovascular risk calculators were absent Combining former smokers with never-smokers might bias outcomes; detailed smoking histories could alter model accuracy Larger datasets and further clinical validation are necessary to evaluate retinal images as potential cardiovascular risk indicators
7	Automated Identification of Diabetic Retinopathy Using Deep Learning (107)	675	A DL algorithm for automated DR detection in retinal images achieved a 0.97 AUC, with 94% sensitivity and 98% specificity. Its efficiency in screening and identifying DR cases for ophthalmologist referral may greatly decrease global DR-related vision loss rates	The method efficiently detected DR without manual grading, reducing costs It was operable on standard personal computers and smartphones, enhancing accessibility Abnormalities were accurately visualized, supporting clinical confirmation It effectively identified early DR stages, highlighting microaneurysms and hemorrhages This approach has the potential to optimize retinopathy screening, reducing vision loss from DR and improving global clinical management	The algorithm struggled to differentiate between healthy and very early cases of DR, particularly those with only a few fine microaneurysms The effect of geographic variations on performance, like retinal pigmentation and DR prevalence, warrants more study Future research should consider integrating patient metadata into the model to improve accuracy and elucidate non-imaging DR risk factors
8	Pivotal trial of an autonomous AI-based diagnostic system for detection of diabetic retinopathy in primary care offices (108)	617	The AI diagnostic system exhibited high sensitivity (87.2%) and specificity (90.7%) for diabetic retinopathy detection in primary care, earning FDA approval. It holds promise for enhancing early DR and diabetic macular edema identification	The system outperformed ophthalmologists in detecting DR among diabetics It accurately identified all severe and proliferative DR cases Exhibited low ethnic or racial bias, enhancing its reliability Design based on lesion detection and clinical insights minimized bias Maintained effectiveness despite cataracts, recommending when dilation is needed for scalability IDx-DR became the first autonomous diagnostic AI to receive FDA approval in medicine	The study has a limited scope, primarily focusing on the efficacy of the AI system in detecting DR and DME The system overlooked incidental findings, including optic disk cup enlargement and signs of retinal pigment epithelium atrophy, suggesting possible conditions like glaucoma or AMD In primary care settings, the AI system’s sensitivity was reduced compared to lab datasets
9	Segmenting Retinal Blood Vessels With Deep Neural Networks (109)	606	The study utilized a supervised deep neural network for retinal vessel segmentation in fundus images, autonomously transforming raw pixels into sophisticated features, demonstrating deep learning’s effectiveness in medical imaging without prior domain knowledge	The method showed resilience against central vessel reflex, highlighting its robustness in fundus imaging It exhibited high sensitivity (>0.87) in detecting fine vessels By converting raw pixels into abstract features, the network enhanced vessel segmentation Utilizing structured prediction, it simultaneously classified pixels, aiding in anatomical structure segmentation	Deep neural networks face criticism for their interpretability issues, obscuring their decision-making process in segmentation tasks The algorithm requires further refinement for widespread clinical adoption
10	Improved Automated Detection of Diabetic Retinopathy on a Publicly Available Dataset Through Integration of Deep Learning (110)	581	The enhanced deep-learning algorithm IDx-DR X2.1 surpassed traditional models in detecting referable diabetic retinopathy (rDR), achieving 96.8% sensitivity and 87.0% specificity, alongside high negative predictive values, demonstrating its accuracy in identifying the absence of DR	The device achieved 100% sensitivity and 91% specificity for vision-threatening diabetic retinopathy It exhibited a high Negative Predictive Value (NPV), indicating a minimal chance of DR and offering patient reassurance Research on the Messidor-2 dataset with a three-expert standard ensured result transparency and reliability	The study, focusing on a standardized dataset, lacks evaluation of the device’s real-world performance in diabetic populations, where variations in image quality and demographics could affect results It did not address the device’s ethical, legal, and financial implications crucial for clinical use

### 3.7 Top 5 retinal diseases researches using AI

We manually screened the keywords from CiteSpace statistics and screened out the keywords for retinal diseases. Furthermore, we refined the search results on using AI in retinal disease on a more granular level using WOS analysis Citation Topics Micro and selected from over 2,500 available micro-level citation topics based on the search results. The “Citation Topic Micro” feature in (WoS) allows researchers to analyze and explore citation patterns at a granular level within a specific topic or field of study. It helps identify the most influential literature, authors, or institutions within a given research area based on their citation impact. This feature facilitates in-depth bibliometric analysis and aids in assessing the influence and significance of scholarly articles within a specific research domain.

The data from the two databases above were statistically analyzed (Table 6). Among the statistical keywords, DR was the most studied disease, followed by Glaucoma. These retinal diseases have mainly been studied and applied to AI.

**Table 6:** Top five retinal disease researches using AI from 2014 to 2023.

Rank	Topic	Times
1	Diabetic retinopathy (DR)	582
2	Glaucoma	428
3	Age-related macular degeneration	384
4	Diabetic macular edema	299
5	Retinopathy of prematurity (ROP)	79

## 4 Discussion

### 4.1 Principal results

From 2014 to 2017, the article counts on the application of AI in retinal diseases showed modest growth. However, a pivotal shift occurred post-2017, sparked by a groundbreaking study by Gulshan et al. from Google Health (43), which demonstrated an automated algorithm capable of accurately interpreting retinal images. This development, characterized by high accuracy and the algorithm’s impressive sensitivity (99% [87.0–97.5%]) and specificity (90.3–98.1%) in diabetic retinopathy screening, fueled advancements in deep learning algorithms for diagnosing and treating retinopathy across diverse populations.

The distribution of publications reveals distinct trends in both output and impact across different countries. China leads in volume, contributing approximately 32% of the global articles. Conversely, the United States boasts the highest h-index at 66, indicating the significant impact of its contributions, followed closely by China with a score of 62. England, with the highest centrality score (0.24) and India following closely (0.22), highlights the importance of international collaborations that strengthen research quality and scope. Notably, joint publications between the United States and China represent 55% of the total output, underscoring the high caliber of collaborative work. Despite this, China’s relatively lower level of international collaboration, especially compared to the active partnerships among the United States, England, and India, suggests an opportunity for China to engage more in transnational research efforts, which could further enhance the global impact of its research.

The global academic landscape features prominent contributions from the UK and China, with these nations housing the majority of the top 10 institutions in this field. China’s recent surge in publications aligns with recent science and technology policy advancements, though its development timeline indicates the need for further consolidation of foundational knowledge. In contrast, the United States’ robust AI ecosystem, supported by research experts and technology firms, plays a crucial role in advancing AI research and applications. This disparity highlights the need for China to enhance its technology development and for the United States to foster greater academic collaborations.

Within Singapore, the National University of Singapore has emerged as a key player in scientific research, demonstrating strong collaborative engagements. On the other hand, the University of London emerges with a broader impact evidenced by its published literature. Alongside, two other UK-based institutions also boast high h-index values. Despite their varied publication frequency and centrality, these institutions collectively mark a significant advancement in academic pursuit, reflecting growth in scholarly output and consistent dissemination of research articles. Analysis of the cooperation network shows that research institutions in this specialty generally have low intermediary centrality, suggesting the need to bolster their influence in the realm. Enhanced collaboration among these institutions is paramount, as insufficient networking can hinder the progression of the domain, potentially impeding academic research.

In the context of AI applications in ophthalmology, an integrative approach combining foundational ophthalmic disease knowledge with innovative computer science methodologies is essential. In Table 6, the top five keywords of retinal disease indicate that DR is the most commonly used AI in fundus diseases, followed by Glaucoma, AMD, and other retinal diseases. These focus areas underscore the importance of AI-driven diagnostic tools for addressing the global burden of retinal diseases and advancing early detection and intervention strategies.

### 4.2 Research hotspots and emerging trends

The current research frontiers in AI application within retinal disease spotlight three main areas: “OCT,” “Attention Mechanism,” and “Outcome.” These fields have been increasingly active from 2021 to 2023 as per the clustering timeline derived from Figure 5, underscoring evolving research directions and technologies.

#### 4.2.1 Optical coherence tomography

Occupying a pivotal role in ophthalmology, OCT is a non-invasive technique that generates high-resolution, cross-sectional imagery of the retina, optic nerve, and anterior eye segment. It’s essential for the detailed analysis and identification of ocular pathologies through OCT fundus images. OCT’s capability to illuminate microstructural details in the eye facilitates the early identification, diagnosis, and tracking of a variety of eye diseases including age-related macular degeneration (44, 45), diabetic retinopathy (46), glaucoma (47), among others (48).

##### 4.2.1.1 Advancements in AI-enhanced OCT for precise retinal analysis

AI integration with OCT has revolutionized the detection and analysis of retinal structures. OCT uses interference patterns to provide detailed images crucial for identifying eye diseases. AI notably enhances segmentation precision, aiding the delineation of ophthalmic microstructures. For instance, He et al. (49) achieved a Dice score of 91.3% and a mean Intersection over Union (mIoU) of 84.4% for retinal layer segmentation. Other studies, such as those by Lu et al. (50) and Guo et al. (51), have demonstrated significant advancements in efficient segmentation with minimal labeled data and automated segmentation of specific retinal features. Overall, AI’s integration with OCT improves fundus lesion detection capabilities. Zhang et al. (52) introduced X-Net, a weakly supervised DL framework for segmenting paracentral acute middle maculopathy lesions in spectral-domain OCT (SD-OCT) images, achieving 99% accuracy and a mIoU of 0.8. Subsequently, Zhang et al. (53) developed RC-Net for segmenting hyperreflective dots in retinal OCT images, essential for DR diagnosis, with a mean Dice similarity coefficient of 75.29% ± 0.42%, an IoU of 62.27%, recall of 78.36%, and precision of 75.34%. Lastly, Loo et al.’s (54) DL algorithm for classifying and segmenting retinal cavitations in macular telangiectasia type 2 via OCT images reported a sensitivity of 0.94, a specificity of 0.80, and an average Dice similarity coefficient of 0.94. Overall, AI’s integration with OCT improves fundus lesion detection capabilities.

##### 4.2.1.2 Advancements in AI-assisted OCT for detection of retinal pathologies

Advanced deep learning algorithms enhance AI’s ability to analyze OCT imagery, significantly improving the identification of subtle retinal and optic nerve abnormalities. The integration of AI with OCT diagnostic processes boosts efficiency and promotes timely intervention, significantly improving patient outcomes.

##### 4.2.1.3 Advancements in AI-assisted OCT for multiple retinal diseases detection

Prevailing AI applications in retinal disease diagnosis have evolved from targeting single conditions to addressing multiple retinal diseases concurrently within the same patient. This shift is facilitated by deep learning algorithms that benefit from larger and better-quality datasets, feature fusion advancements, and holistic training techniques. This new wave of multi-condition AI detection models has quickly gained traction in academic research. A prime example is Sunija et al. (55), who introduced OctNET, a lightweight CNN for classifying retinal diseases via OCT images, achieving precision, recall, and accuracy rates of 99.69%. Togacar et al. (56) proposed a novel approach combining CNN-derived dominant activations with the slime mold algorithm, resulting in high classification accuracies of 99.60, 99.89, and 97.49% across three distinct datasets. Similarly, Upadhyay et al. (57) presented a CNN framework for four-class retinal disease classification, incorporating batch normalization across five layers to attain a 97.19% accuracy rate. Kayadibi et al.’s (58) Retinal Fine Tuned Convolutional Neural Network (R-FTCNN) demonstrated perfect metrics in one dataset in the Duke dataset and exceptional results in the UCSD dataset, with an accuracy of 99.70%, a sensitivity of 99.70%, and a specificity of 99.90%.

The Hercules model integrates attention mechanisms and uncertainty quantification to enhance classification accuracy, achieving 94.21% in retinal OCT evaluations (59).

Moreover, AI’s potential to revolutionize diagnostic methods in universal healthcare systems, especially in community settings, is increasingly recognized. Bai et al. (60) assessed AI-enabled OCT’s precision in identifying 15 retinal disorders in community environments, demonstrating performance comparable to retina specialists and surpassing both senior and junior ophthalmologists. This suggests AI’s capability to improve accessibility and quality in community-based ophthalmic healthcare.

##### 4.2.1.4 Advancements in AI-assisted OCT for DR

The research community is keenly focused on utilizing AI with OCT to explore DR-related retinopathy, with particular attention to microangiopathy. OCT’s capability in detecting subtle microvascular alterations serves as a cornerstone for early DR detection. Deep learning models have substantively aided clinicians in identifying early-stage DR using OCT. Hua et al. (61) introduced TFA-Net, a DL framework for assessing DR severity through fundus images and wide-field swept-source OCT angiography (SS-OCTA), achieving a Quadratic Weighted Kappa (QWK) of 90.2% in the KHUMC dataset and a mean accuracy of 94.8% with an AUC of 99.4% in the Messidor dataset.

OCT is also vital for detecting and assessing diabetic macular edema (DME), enabling high-resolution imaging that identifies abnormal retinal thickening and specific morphological changes associated with DME. DME is categorized as central involvement (CI-DME) or non-central involvement (NCI-DME) (62). Tang et al. (63) developed DeepDR, a DL framework for diagnosing and staging DME, which achieved AUROC scores of 0.937, 0.958, and 0.937 on the CIRRUS, SPECTRALIS, and Triton OCT datasets, respectively, and 0.965 on an external dataset. In differentiating between CI-DME and NCI-DME, AUROC results were 0.968, 0.951, and 0.975 for the primary dataset, exceeding 0.894 for the external dataset, highlighting DeepDR’s effectiveness in DME assessment.

Additionally, DL techniques enhance OCT’s capability to reveal characteristic changes indicative of DME severity, treatment response, and prognosis, making it a valuable imaging biomarker (64, 65). A study on the automated quantification of central macular fluid volume (CMFV) via OCT involved 215 patients, finding an AUROC of 0.907 for identifying center-involved DME. With a specificity of 95%, CMFV demonstrated a sensitivity of 78.5% for detecting center-involved DME, confirming its role as a diagnostic biomarker (66). Further research has underscored a significant correlation between the extent of the area of avascularity (EAA) measured via OCTA and DME severity. Notably, EAA’s measurements showed no significant correlation with factors like signal strength index and shadow area, affirming its reliability and independence as a biomarker for DME (67).

##### 4.2.1.5 Advancements in AI-assisted OCT for AMD diagnosis and segmentation

Age-related macular degeneration (AMD) has a long developmental cycle and variable symptom progression, necessitating AI algorithms that effectively address disease classification and staging. Accurate training methods are crucial for these applications, as OCT image changes are indicative of disease progression. Thomas et al. (68) introduced a pioneering deep CNN architecture tailored for the early detection of AMD from OCT images, showcasing high accuracy with scores ranging from 0.9666 to 0.9978 across multiple datasets. Moradi et al. (69) explored deep ensemble learning for automated classification of non-advanced AMD, utilizing optimized retinal layer segmentation in spectral-domain OCT (SD-OCT) scans, resulting in an AUC of 99.4% for distinguishing normal eyes from early AMD cases. Sotoudeh-Paima et al. (70) developed a multi-scale CNN for automated AMD classification, implementing gradual learning stages that enhanced accuracy from 87.2 to 93.4%. Liefers et al. (71) developed and validated a deep learning (DL) model for identifying features of neovascular and atrophic AMD in OCT images, achieving a mean Dice score of 0.63 for 11 of 13 features and an intraclass correlation coefficient of 0.66, both comparable to human observers.

##### 4.2.1.6 Advancements in AI-assisted OCT for diagnosis and grading of glaucoma

As can be seen from above, advancements have been achieved in DL methods for assessing changes in cup-to-disk ratio (C/D ratio) and retinal nerve fiber layer (RNFL) thickness. Over recent years, substantial progress has been made in leveraging DL for assisted diagnosis and grading of glaucoma using OCT imaging.

For glaucoma detection, Metha et al. (72) developed a multimodal DL model that combines macular OCT volumes, color fundus photographs, and clinical data for glaucoma detection, achieving an AUC of 0.97 with data from the UK Biobank. Sun et al. (73) introduced a dual-input CNN that analyzes RNFL and ganglion cell-inner plexiform layer (GCIPL) images, demonstrating strong diagnostic performance with an accuracy of 92.793% and an AUC of 0.957. Panda et al. (74) investigated the diagnostic utility of the central retinal vessel trunk and branches’ 3D structure for glaucoma using OCT, employing a DL network for segmentation, achieving a Dice coefficient of 0.81. Utilizing 3D and 3D-to-2D CNN methodologies, they discerned glaucoma from non-glaucoma cases with accuracies of 82.7 and 83.3%, and AUCs of 0.89 and 0.90, respectively. In a study analyzing 130 eyes from healthy subjects and 275 eyes from individuals diagnosed with glaucoma, CNN was utilized to evaluate vessel density and RNFL thickness images, achieving an Area Under the Precision-Recall Curve (AUPRC) of 0.97 (75). Beyond traditional methods, He et al. (76) presented a modality-specific attention network that integrates fundus and OCT images for retinal image classification, highlighting the advantage of multi-modal data integration. For glaucoma grading, Garcia et al. (77) proposed a hybrid network combining hand-crafted features with DL algorithms for analyzing circumpapillary B-scans. This approach, utilizing prototypical networks for few-shot learning, achieved categorical accuracies up to 0.9459.

In addition, AI has the capability to synthesize OCT images to aid in training AI systems for glaucoma diagnosis. Sreejith Kumar et al. (78) evaluated the use of Generative Adversarial Networks (GANs) to train DL networks on synthetic images, achieving an AUC of 0.97 on the internal dataset and 0.90 on the external dataset. These results demonstrate the efficacy of generative models in DL training and the potential for data sharing across institutions.

#### 4.2.2 Attention mechanism

The attention mechanism is a technique in DL that enhances model’s focus on specific input data, thereby optimizing its performance. The articles with the keyword “attention mechanism” show its widespread application in medical image analysis, especially in DR classification. In retinal diseases, attention mechanisms are crucial for lesion detection, diagnosis, and classification. Here are some key points and research directions related to attention mechanisms in the context of the included articles.

##### 4.2.2.1 Retinal structure segmentation based on CFP

In the realm of fundus image segmentation within the domain of AI, the attention mechanism technology integrated within CNNs emerges as a pivotal advancement, directing the model’s focus toward pertinent regions within retinal images. This sophisticated technique has garnered considerable attention and application, particularly in the segmentation of fundamental anatomical features such as blood vessels and optic disks, yielding commendable outcomes.

In vascular segmentation, this method has achieved remarkable achievements. Notable techniques include the AACA-MLA-D-UNet, employing a multi-level attention mechanism and dropout dense blocks for enhanced segmentation accuracy, showing superior performance on DRIVE, STARE, and CHASE_DB1 datasets with reduced complexity (79). Li et al. (80) improved U-Net-based retinal vessel segmentation by incorporating attention mechanisms, yielding better results across multiple datasets. BSEResU-Net utilizes Before-activation Squeeze-and-Excitation blocks (BSE Blocks) with attention mechanisms and drops block regularization to improve performance and generalization. Experimental results on DRIVE, STARE, and HRF datasets with F1-scores of 0.8324, 0.8368, and 0.8237, respectively (81). MSCNN-AM, a multi-scale CNN incorporating attention mechanisms for retinal vessel segmentation, demonstrated notable efficacy, with sensitivities of 0.8342/0.8412/0.8132 and accuracies of 0.9555/0.9658/0.9644 on DRIVE, STARE, and CHASE_DB1, respectively (82). A novel retinal vessel segmentation algorithm, integrating multi-scale attention D-MNet with an enhanced PCNN model, was validated across four databases (DRIVE, STARE, CHASE_DB1, HRF), achieving detection accuracies of 96.83, 97.32, 97.14, and 96.68%, respectively (83). LEA U-Net, a DL framework for retinal vessel segmentation has an evaluation on the DRIVE dataset achieving an accuracy of 0.9563, F1-score of 0.823, TPR of 0.7983, and TNR of 0.9793. The AUC of PRC is 0.9109 and the AUC of ROC is 0.9794 (84).

Besides, the utilization of attention mechanism technology within CNNs has demonstrated remarkable efficacy in segmenting critical ocular structures such as the optic disk and cup, pivotal in glaucoma screening protocols. Guo et al. (85) established FAU-Net, enhancing U-Net with feature fusion and channel-spatial attention, showing superior performance across multiple datasets. RSAP-Net introduces a U-shaped network with a Residual Spatial Attention Path for optic disk (OD) and optic cup (OC) segmentation, achieving F1 scores of 0.9752 (OD) and 0.9012 (OC), and boundary localization errors of 6.33 pixels (OD) and 11.97 pixels (OC) on Drishti-GS1 (86). Wang et al. (87) created a hierarchical CNN with a cascaded two-stage architecture for OD and OC segmentation in fundus images, utilizing an attention mechanism and focal loss for accurate OD identification, followed by multi-task and adversarial learning for OD and OC segmentation. The model exhibited competitive performance on RIM-ONE-r3 and REFUGE datasets.

##### 4.2.2.2 Diagnosis, classification, and staging of DR

DR classification and staging based on DL have seen significant advancements in recent research. According to the American Academy of Ophthalmology’s international clinical classification system for diabetic retinopathy (DR), DR is categorized into various stages, including no DR, mild non-proliferative DR (NPDR), moderate NPDR, severe NPDR, proliferative DR, and diabetic macular edema (DME). Several studies have contributed novel DL models to enhance DR grading accuracy and performance. He et al. (88) introduced CABNet, a CNN with an attention module, achieving enhanced lesion detection and handling of imbalanced data, achieving accuracies of 78.98, 84.08, and 86.18%, with Kappa scores of 0.7863, 0.8723, and 0.8678 on the DDR, Messidor, and EyePACS datasets, respectively. Papadopoulos et al. (89) proposed an interpretable method with an attention mechanism for referable DR detection, with AUCs of 0.961 in Kaggle and 0.976 in Messidor-2, and with valid lesion heatmaps with AUPRC of 0.869 in IDRiD. Li et al. (90) established DACNN, an attentive CNN for imbalanced DR grading in retinal images, incorporating attention mechanisms for improved feature extraction with the results of 88.0% accuracy and 88.6% kappa score for multi-class DR grading on the EyePACS dataset, and 98.5% AUC, 93.8% accuracy, 87.9% kappa, 90.7% recall, 94.6% precision, and 92.6% F1-score for referral and non-referral classification on the Messidor dataset. Jian et al. (91) created Triple-DRNet, a three-stage cascade network with attention-enhanced subnets, achieving 92.08% accuracy and a 93.62% Quadratic Weighted Kappa. This model surpassed previous networks with accuracies of 98.99% for DR-Net, 88.40% for PDR-Net, and 80.20% for NPDR-Net. Murugappan et al. (92) implemented a Few-Shot Learning network with an attention mechanism for DR detection and grading, performing well both in detection (99.73% accuracy, 99.82% sensitivity, 99.63% specificity) and in grading (98.18% accuracy, 97.41% sensitivity, 99.55% specificity). Reddy and Gurrala (93) developed the Joint DR-DME Network (JDD-Net), which utilizes a deep graph correlation learning model. The model combines convolutional block attention module (CBAM) and joint disease attention (JDA) modules to extract disease-specific features, attaining accuracies of 99.53% for individual DR, 99.1% for individual DME, and 99.01% for combined DR-DME grading. In addition to traditional CNNs, Wu et al. (94) employed a Vision Transformer for DR grading, leveraging attention mechanisms for long-range pixel analysis, resulting in 91.4% accuracy, 97.7% specificity, 92.8% precision, 92.6% sensitivity, a QWK of 0.935, and an AUC of 0.986.

However, a common issue persists: these DL systems for DR grading often lack integration with medical knowledge. Consequently, ophthalmologists face challenges in accurately interpreting grading outcomes, limiting the practical applicability of these systems. To address the problem, Tian et al. (95) applied FA + KC-Net to integrate medical knowledge into DR grading, enhancing interpretability for ophthalmologists. The model achieved 84.49% accuracy and 86.17 QWK across datasets.

#### 4.2.3 Outcome

AI has a high reference value for assessing the prognosis of retinal diseases. Accurate prediction will help physicians design treatment plans, improve the quality of care, and improve patient outcomes. Hashimoto et al. (96) trained a CNN with data from 591 eyes to predict visual field (VF) sensitivity using macular layer thickness, achieving low absolute errors (AE of 2.84 dB for the entire VF). This DL model also accurately predicted VF outcomes from the HFA 10–2 test using SD-OCT data. Li et al. (97) analyzed data from 17,497 eyes to develop and validate DL models for glaucoma forecasting, achieving AUROCs of 0.90 for incidence and 0.91 for progression in the validation cohort. External tests showed AUROCs of 0.87 to 0.89 for incidence and consistently 0.88 for progression, demonstrating robust generalizability. Yang et al. (98) utilized an automated algorithm to assess OCTA images from diabetic patients over 4 years, revealing that diabetic microvascular infarctions (DMIs) in both superficial and deep capillary plexuses signal diabetic retinal disease progression. Specifically, DMIs in the deep capillary plexus were significantly associated with DME onset and visual acuity deterioration.

##### 4.2.3.1 Prediction of vision results

A significant advance in the treatment of retinal illnesses is intravitreal anti-vascular endothelial growth factor therapy; however, its effect on some patients is undesirable. AI technology can accurately predict the individual effects of treatment. Ideally, after the initial treatment, the AI model would take baseline photos and clinical data of the patient and provide anticipated follow-up intervals and overall treatment expectations. This could significantly improve the planning of anti-vascular endothelial growth factor therapy, prevent negative effects caused by under-treatment or over-treatment, and reduce treatment costs. Seebock et al. (99) used the random forest AI model to train the prognosis of patients receiving standardized ranibizumab treatment, and the accuracy rate of predicting individual vision results was 71%.

##### 4.2.3.2 Prediction of the future natural course of retinal diseases

Early AMD is a chronic progressive disease with a highly heterogeneous progression rate. Patients with AMD may remain in the early stages without any associated functional impairment and rapidly progress to advanced AMD. Predicting advanced AMD is difficult in clinical practice. Ajana et al. (100) developed a machine-learning model for advanced AMD prediction, incorporating various risk factors and achieving a cross-validated AUC of 0.92 in training and test sets over 5 years, using data from two cohort studies. Moraes et al. (101) introduced a DL algorithm for automated OCT scan quantification in neovascular AMD, utilizing data from the Moorfields Eye Hospital AMD Database. This algorithm segmented features such as neurosensory retina and fluids, noting differences in feature volumes between first and second-treated eyes and among demographic groups, highlighting automated OCT segmentation’s potential in personalizing care and uncovering new structure–function relationships.

### 4.3 Open challenges and future opportunities

Summarizing the limitations of the research on the application of AI in retinal disease can guide future research. The limitations of the citations can be roughly divided into five aspects: (1) language limitations in the groups included in the studies; (2) Source limitations of the selected articles; (3) limitations of research methods; (4) bias caused by the subjective will of the participants; and (5) limitations of small sample sizes.

To overcome the problem of limitations in the groups included in the studies, researchers should aim to increase the diversity of the study sample by including participants with a wider range of characteristics, such as age, gender, race, ethnicity, socioeconomic status, and health status. This will help to ensure that the results are applicable to a broader population. To address the problem of limitations of research methods, researchers should consider using multiple methods to collect data, verifying their findings by replicating the study, 5 or using complementary methods. To mitigate the bias caused by the subjective will of the participants, researchers can use various strategies, such as ensuring informed consent, using blinding techniques, and avoiding leading questions. To address the limitations of small sample sizes, researchers can try to increase the sample size by recruiting more participants or by using statistical methods to compensate for the smaller sample size. It is also important to report effect sizes and confidence intervals to provide a more accurate estimate of the strength and precision of the results.

From the perspective of hot research directions, there is still room for improvement in future research. The dataset standard is inconsistent. Many retinal diseases show similar manifestations; the diagnosis mainly relies on experienced doctors, and there are variations in diagnosis between doctors. Given this situation, many research and development teams have collected several fundus pictures of different eye diseases, invited senior ophthalmologists to read the pictures, graded and partitioned the pictures, and established diagnostic datasets for some common diseases. However, many diseases are still without officially recognized datasets, and various reliable and unreliable data sets limit current diagnosis and treatment efforts.

Although many scholars have carried out some research work in this field and achieved phased results, the application of AI in retinal diseases mainly focuses on DR, AMD, and glaucoma at present, more data accumulation and data mining is still needed for retinal diseases such as ROP and myopia. In the future, Relevant researchers should develop more systematic prediction models, detect the hidden information in the images according to the guidance of clinicians, incorporate more evaluation factors, and conduct multimodal analysis to predict the treatment progress and success probability of retinal diseases.

### 4.4 Limitations

Our study encountered several limitations that merit consideration. We confined our literature review to articles dated between 2014 and 2023. Moreover, our search was restricted to English-language literature, thereby excluding potentially valuable data from unpublished or in-progress works in other languages. Additionally, our analysis was confined to the Web of Science Core Collection (WoSCC), a recognized academic database. While incorporating other databases could broaden the dataset, variations in citation metrics and indexing methods across platforms complicate cross-database integration and analysis. Furthermore, limitations in the literature screening process, stemming from both automated tools and manual exclusion, may have introduced minor inaccuracies. Finally, although we rigorously examined 4,386 articles, the study may still reflect a degree of inherent researcher bias that is challenging to eliminate entirely.

## 5 Conclusion

Through bibliometric analysis of worldwide literature over the past decade, we have traced the historical evolution, pinpointed current research foci, and projected future trends within this field. Despite progress, there remains a pressing need for refinement of DL algorithm models and enhancement of disease process screening methods. China holds the record for the highest volume of literature published, whereas the United States stands out as the most influential contributor to this research area, with Germany leading in international collaborations. The University of London and the National University of Singapore are distinguished by the high citation rates of their articles.

The refinement of AI algorithms and the exploration of abnormal eye physiological features constitute core pursuits in AI-assisted diagnosis of retinal diseases. Looking ahead, research should pivot toward developing sophisticated diagnostic systems for ophthalmic diseases and fostering integration between computer engineering and ophthalmology. Current challenges, such as bringing products to market, the lack of clinical validation, and data standardization issues, all require urgent attention. Furthermore, adapting to the dynamic landscape of medical knowledge is crucial.

Current challenges—including the need for market-ready products, clinical validation, and data standardization—require urgent attention. Adapting to the dynamic landscape of medical knowledge is also crucial. We recommend harnessing AI technology to support mobile healthcare initiatives, the development of intelligent health devices, and the real-time monitoring and evaluation of individual health. These advancements will not only facilitate early disease detection, DR screening, and proactive interventions but will also provide patients suffering from retinal diseases with safer, more convenient, and vastly improved management services.

## Data Availability

Publicly available datasets were analyzed in this study. This data can be found here: https://webofscience.clarivate.cn/wos/woscc/basic-search.
